# ASLNet: an explainable deep learning framework for glioma grading and survival prediction

**DOI:** 10.3389/fonc.2026.1818663

**Published:** 2026-05-18

**Authors:** Rafail C. Christodoulou, Georgios Vamvouras, Platon S. Papageorgiou, Evros Vassiliou, Sokratis G. Papageorgiou, Christina Kalogeropoulou, Peter Zampakis, Elena E. Solomou, Michalis F. Georgiou

**Affiliations:** 1 Division of Neuroimaging and Neurointervention, Department of Radiology, Stanford University, Stanford, CA, United States; 2 Department of Electrical and Computer Engineering, National Technical University of Athens NTUA, Athens, Greece; 3 Department of Medicine, National and Kapodistrian University of Athens, Athens, Greece; 4 Department of Biological Sciences, Kean University, Union, NJ, United States; 5 1st Department of Neurology, Medical School, National and Kapodistrian University of Athens, Eginition Hospital, Athens, Greece; 6 Department of Radiology, University of Patras Medical School General Hospital of Patras, Patras, Greece; 7 Internal Medicine-Hematology, University of Patras Medical School, Patras, Greece; 8 Department of Radiology, University of Miami, Miami, FL, United States

**Keywords:** arterial spin label (ASL) MRI, artificial intelligence, deep learning, gliomas diagnosis, grade prediction, survival & prognosis

## Abstract

**Introduction:**

Arterial spin labeling (ASL) MRI provides noninvasive quantitative perfusion information and may capture vascular heterogeneity associated with glioma aggressiveness and prognosis. Deep learning (DL) approaches applied directly to ASL volumes may therefore support imaging-based prediction of tumor grade and survival. This study aimed to develop and validate ASLNet, an interpretable three-dimensional residual network framework trained on ASL MRI to predict histopathologic grade and overall survival (OS) in patients with diffuse glioma.

**Methods:**

This retrospective study included 471 patients with histologically confirmed diffuse glioma who underwent ASL MRI between 2015 and 2021. Original three-dimensional ASL volumes were used as model inputs, together with isotropically resampled, Gaussian-smoothed, and z-score-normalized versions. Two custom three-dimensional residual networks were trained: one model for WHO grade classification and a second FiLM-type intermediate-fusion model incorporating ASL imaging with demographic and clinical variables, including age, sex, and extent of resection, for OS classification (<12 months vs. ≥12 months). Model performance was evaluated on held-out test sets using area under the receiver operating characteristic curve (AUC), macro-F1 score, accuracy, and recall. Integrated gradients were used to generate saliency maps and identify influential perfusion regions.

**Results:**

The ASLNet grading model achieved an AUC of 0.79, macro-F1 score of 0.74, accuracy of 0.60, and recall of 0.73. The OS prediction model achieved an AUC of 0.70, macro-F1 score of 0.73, accuracy of 0.66, and recall of 0.94 for the long-survival class. Saliency analysis highlighted hyperperfused tumor cores as influential regions for grade prediction, while survival prediction also involved peritumoral regions, consistent with biologically plausible patterns of perfusion heterogeneity in gliomas.

**Discussion:**

ASLNet demonstrates the feasibility of interpretable, perfusion-based deep learning for glioma grade and survival prediction using ASL MRI. These findings suggest that ASL contains clinically relevant information related to tumor vascular characteristics and prognosis. Although further external validation is warranted, this approach supports the potential clinical value of ASL-based DL as a complementary noninvasive tool for glioma risk stratification.

## Graphical Abstract

**Figure:**
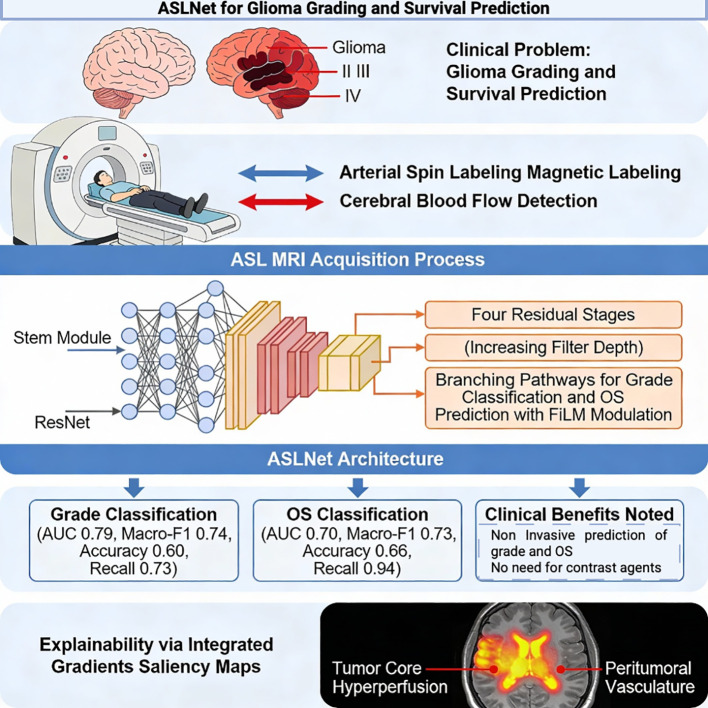
Created in BioRender. Christodoulou, R. (2026) https://BioRender.com/3su4za2.

## Introduction

Accurate preoperative glioma grading remains a crucial clinical task, serving as a foundation for patient management by guiding surgical decisions and adjuvant therapy, and influencing patient outcomes and prognosis (1). Differentiating high-grade gliomas (HGG) from low-grade gliomas (LGG) enables clinicians to design appropriate treatment plans and counsel patients on disease prognosis (2). Undoubtedly, tumor grade is strongly linked to disease outcomes, with median survival rates for grade II sometimes exceeding 10 years, whereas glioblastoma (grade IV) rarely surpasses 14 months (3, 4). In clinical practice, definitive glioma grading continues to rely on intracranial biopsy, which is invasive, carries procedural risks, and may lead to diagnostic errors (5). Since gliomas, especially HGGs, exhibit significant heterogeneity within tumors, a small biopsy sample may not capture the full pathological landscape, leading to sampling error and misclassification (6, 7). Therefore, non-invasive imaging for glioma grade assessment has been studied prior to resection, as it appears to influence treatment planning, prognosis, and overall patient care (8). The latest WHO glioma classification further emphasizes the importance of accurate preoperative diagnosis to optimize therapy in patients with glioma (1).

Perfusion MRI techniques play a crucial role in evaluating the vascularization and aggressiveness of gliomas. HGG promotes neoangiogenesis and requires increased blood supply, making tumor blood flow and microvascular density valuable indicators of tumor grade (9, 10). Advanced perfusion imaging techniques have been explored to capture these differences in tumoral vascular heterogeneity. Dynamic susceptibility contrast (DSC) MRI is currently the most widely used and clinically established perfusion technique in neuro-oncology, owing to its relatively high signal-to-noise ratio, reduced susceptibility to blood flow variations, and the extensive validation of relative cerebral blood volume (rCBV) measurements in brain tumor imaging (11). However, it requires gadolinium-based contrast administration and can be affected by susceptibility artifacts, contrast leakage, and post-treatment blood products, such as microhemorrhages (12). ASL is an advanced perfusion MRI method that uses magnetically labeled arterial water as an endogenous tracer, enabling quantitative mapping of cerebral blood flow without external contrast agents. In neuro-oncology, ASL is attractive because it has shown promising results in indicating tumor perfusion, strongly correlates with vessel density in gliomas, and can effectively distinguish between LGG and HGG. ASL adds only a few minutes to an MRI exam, carries no risk of contrast-related adverse reactions, and offers an accurate assessment of tumoral perfusion (13). Therefore, it could be a valuable addition to conventional MRI sequences, providing essential insights into tumor biology.

Emerging DL approaches have been applied to brain tumor MRI to automate diagnosis and prognosis (14).A convolutional neural network (CNN) can capture complex imaging patterns in glioma imaging that may otherwise be overlooked by the human eye. The combination of multiple MRI sequences (T1, T2, FLAIR) enables the model to leverage information from different tumor components, such as the enhancing portion and necrosis, thereby improving classification accuracy (15). Prior studies have successfully presented DL frameworks for glioma segmentation, grading, and survival prediction. Sun et al. developed a 3D CNN ensemble model based on majority voting for glioma segmentation, combined with a radiomics-based random forest to predict overall survival (16). These applications underscore the potential of DL applications in gliomas and highlight the expanding role of DL in neuro-oncology imaging.

Despite promising results, a gap remains in the current artificial intelligence (AI) literature for glioma imaging. A significant challenge is the lack of interpretability, which often turns most frameworks into “black boxes” and undermines clinicians’ trust, especially in critical tasks such as predicting glioma grade or prognosis. This is because radiologists cannot access the factors influencing the model’s decisions and lack transparent reasoning (14). This has already prompted calls for explainable AI frameworks that enable clinicians to verify that predictive features align with intuitive imaging findings (17). ASL perfusion MRI is rarely used, and models that rely solely on ASL for glioma evaluation are scarce. Since ASL directly measures tumor vascularity, focusing exclusively on perfusion could yield a more biologically relevant and understandable approach. We propose that perfusion patterns derived solely from ASL are sufficient to predict glioma grade and patient survival. ASLNet, an interpretable 3D ResNet framework derived from ASL perfusion and complemented by key clinical variables for survival modeling, aims to address this unmet need by providing a transparent, biologically meaningful tool for non-invasive glioma evaluation. To our knowledge, this is the first explainable DL that leverages ASL perfusion alone to perform both glioma grading and survival prediction.

## Materials and methods

The goal of this study was to assess whether ASL perfusion MRI, analyzed with an interpretable deep learning framework, can provide reliable non-invasive prediction of glioma grade and patient survival before treatment. All code required to reproduce the experiments, including model training and evaluation scripts, is publicly available on the GitHub repository at https://github.com/georgeDV2002/ASL-based-tumor-grade-and-OS-classification-ResNets.

This study was conducted and reported in accordance with the CLAIM (Checklist for Artificial Intelligence in Medical Imaging) guidelines. A completed CLAIM checklist is provided in **Supplementary Table 4**.

### Cohort selection

Five hundred one patients in the UCSF-PDGM cohort with glioma diagnoses were screened. We included patients who met the following criteria: (1) age ≥18 years, (2) histopathologically confirmed WHO grade II–IV diffuse glioma, (3) availability of pre-treatment ASL MRI of sufficient diagnostic quality, and (4) complete clinical annotation, including tumor grade and overall survival. Patients were excluded if they had (1) severely artifactual or non-diagnostic ASL acquisitions or (2) missing key clinical variables. In total, 30 patients were excluded due to severe ASL artifacts, leaving a final cohort of 471 patients for model development and evaluation. Of these, 390 patients had complete 12-month survival information and were therefore included in the OS classification task.

### Image acquisition

MRI data for training, validation, and testing were obtained from the UCSF-PDGM cohort (18). Based on the scanner type and dataset origin, the ASL acquisition is consistent with standard clinical 3D pseudo-continuous ASL (pCASL) protocols used on GE systems, although exact sequence parameters, such as CASL/pCASL implementation, TR, TE, post-labeling delay, or labeling duration, were not available in the public dataset.

### Software and physical system setup

Model development was performed using Python (v. 3.10.18) and TensorFlow (v. 2.15.0) with CUDA (v. 12.9) on an RTX 4080 GPU with 16 GB of RAM.

### Preprocessing

Preprocessing consisted of four steps: ROI definition, feature-channel creation, data augmentation, and the definition of the training and test sets. These steps were identical for both the grade and OS tasks, and only ASL volumes were used. All scans were originally 240×240×155 with 1 mm^3^ isotropic voxels. A predefined, fully automated ROI extraction strategy was applied, in which the tumor region was approximated by selecting the 0.01% brightest ASL voxels, computing their centroid, and extracting a 70×70×70 mm^3^ cube centered on that centroid, without manual or semi-automated intervention. Visual inspection against corresponding T1-weighted post contrast (T1CE) images confirmed adequate tumor coverage, although some background voxels were included in a subset of cases. To mitigate this, a per-scan background threshold $v_{f}$ was estimated by detecting small, low-intensity, uniform subregions (≈0.5%), which were excluded from subsequent statistical analyses. Three ASL-derived feature channels were then generated: robust, background-free standardization using the median and MAD; local standardization within 15×15×15 mm^3^ subregions; and a log-based transform, aimed at enhancing specific aspects of the raw signal and helping the model extract useful information. Augmentation included flips, ± 10° rotations, elastic deformations, and Rician noise, which, contrary to Gaussian noise, accurately reflects the intensity noise distribution. Finally, grade prediction was treated as a ternary classification (WHO Grade II, III, and IV) with patient-level splitting, while OS prediction used the dataset’s binary 12-month mortality label. More details are available in the supplement under *Detailed Preprocessing Steps*.

### Descriptive statistics

Baseline cohort characteristics are summarized in **Table 1**. All 471 patients had WHO grade available, while 390 had complete 12-month survival information and were therefore included in the OS task. The train-test split for each task was performed at the patient level using stratified sampling to prevent leakage and preserve class distributions. As shown in **Table 1**, the OS task resulted in a balanced split, with nearly identical event prevalence between the TRAIN-CV pool (0.558) and the held-out TEST set (0.551), and no statistically significant differences in survival distribution, age, sex, or extent of resection (all p > 0.60).

**Table 1:** Descriptive statistics.

Variable	Count	Percentage	Variable	Count	Percentage
EOR			WHO grade		
STR	185	39.3%	grade 2	53	11.3%
biopsy	51	10.8%	grade 3	41	8.7%
GTR	234	49.7%	grade 4	377	80.0%
empty	1	0.2%	empty	0	0.0%
Mortality			Sex		
death	231	49.0%	male	279	59.0%
survival	240	51.0%	female	192	41.0%

For the WHO grade task, all patients were retained, but because grade III tumors were rare (8.7%), all grade III cases remained in the TRAIN-CV pool to avoid an empty minority class in the test set. Consequently, the grade-based TRAIN and TEST sets reflect the expected predominance of grade 4 tumors, but do not differ significantly in relative class composition (χ² p = 0.94). Overall, these results confirm that the stratified splitting strategy yielded TRAIN and TEST cohorts that are statistically equivalent for both prediction tasks, minimizing sampling bias and ensuring a fair evaluation of model performance.

In addition, **Supplementary Table 1** provides the full statistical comparison between TRAIN-CV and TEST sets for both OS and WHO grade tasks, including counts, percentages, survival summary statistics, and p-values. No variable differed significantly between partitions (all p > 0.60 for OS and χ² p = 0.94 for WHO grade), confirming that the stratified splitting procedure generated well-balanced, statistically equivalent subsets.

### Explainability

Voxel-level saliency maps for the ASL volume were generated using Integrated Gradients (IG), highlighting each voxel’s contribution to the model’s prediction. Additional details are provided in the *Explainability* section of the supplement. Channel-wise contributions were quantified using exact Shapley values, expressed as sample-wise percentages, and averaged across the test set to characterize the model’s overall behavior.

### Rationale for architecture choice

A 3D ResNet was chosen because the depth required to extract meaningful ASL features, from fine-scale perfusion patterns to broader spatial signatures, demands stable gradient and signal flow. Residual connections prevent vanishing gradients and training instability, while allowing the network to remain sufficiently deep to model glioma heterogeneity. This is essential for ASL, where subtle contrast differences and low SNR require multi-level feature extraction without losing early-layer information. Paired with our centroid-based 70×70×70 mm^3^ ROI, which may include necrotic or non-tumoral tissue, the hierarchical residual structure helps the model suppress irrelevant voxels while preserving informative perfusion cues throughout the network. In glioblastoma, where perfusion varies spatially, this architecture enables robust extraction of volumetric representations even in the presence of noise, necrotic cores, and anatomical variability.

### Grade classification architecture

A 3D convolutional neural network with a ResNet-style architecture is employed, with hyperparameters such as the number of blocks per stage, initial filters, dropout rate, and bottleneck configuration being determined during hyperparameter optimization using Optuna. Inputs of size (70×70×70×4) are first processed by a stem module consisting of a 7×7×7 convolution with stride 2, followed by Group Normalization, ReLU activation, and a 3×3×3 max-pooling operation with stride 2.

Subsequently, four residual stages are applied. Each stage is composed of a configurable number of residual blocks containing 3×3×3 convolutions, Group Normalization, ReLU activations, and optional projection shortcuts. The first block of Stage 1 is applied with a stride of 1, while the first blocks of Stages 2–4 downsample the spatial dimensions using a stride of 2. Filter dimensionality doubles at the transition between stages, enabling progressively deeper feature representations, while skip connections preserve stable gradient flow.

Finally, global average pooling is applied to aggregate spatial information, followed by dropout, and a fully connected, Softmax-activated layer produces class probabilities for the three WHO tumor grades. For a more detailed presentation of the developed architecture, please refer to the *Detailed Grade Classification Architecture* section of the supplement.

### OS classification architecture

The OS classifier uses the same ResNet-based architecture as the grade model, with the addition of incorporating tabular covariates. Age, sex, and EOR are first normalized and passed through a small MLP with dropout, producing two conditioning vectors $\gamma_{c}$, $\beta_{c}$. These are applied to the outputs of the last three residual stages using feature-wise Linear Modulation (FiLM) (19), enabling parameter-efficient and stable modulation of image features. FiLM is restricted to deeper layers, allowing early convolutional blocks to focus on low-level anatomical structure, while clinical variables adaptively influence high-level representations. For a more detailed presentation of the developed architecture, please refer to the *Detailed OS Classification Architecture* section of the supplement.

### Training regime

During training, both tasks use similar procedures. Models are optimized with the AdamW optimizer under Stratified Group K-Fold cross-validation to prevent subject-level leakage, using categorical cross-entropy with label smoothing, with all partitions defined at the patient level to ensure subject-level independence and prevent data leakage. Within each cross-validation fold, a validation subset was used exclusively for early stopping, learning-rate scheduling, and model selection. At the same time, the held-out test set was not accessed during training. Model weights were initialized using the default TensorFlow/Keras initializers, and biases were initialized to zero. Validation performance is monitored using macro one-vs-rest AUC, with early stopping and ReduceLROnPlateau to control convergence and prevent overfitting. In the OS classification task, the model performs *post-hoc* threshold tuning on out-of-fold predictions. In contrast, in the WHO grade classification task, it learns class-specific scaling factors to improve macro-F1. In both cases, the best fold models are restored during inference and are evaluated on the held-out test set.

### Decision curve analysis

Decision Curve Analysis (DCA) was employed to evaluate the clinical utility of the proposed models by quantifying their net benefit across a range of decision threshold probabilities. Unlike performance metrics that assess discrimination or calibration alone, DCA explicitly accounts for the trade-off between true-positive and false-positive decisions across different threshold levels, thereby reflecting the clinical consequences of model-based decision-making. For a given threshold probability $p_{t}$, net benefit was computed using Equation 1, where TP and FP denote the numbers of true-positive and false-positive predictions, respectively, and N is the total number of evaluated samples. The model’s net benefit was compared with two reference strategies: treating all patients (“treat-all”) and treating none (“treat-none”). In this context, treatment refers to taking a clinical action based on a positive prediction from the model. For the grading task, the model’s decision may lead to escalation, either toward histopathologic confirmation or toward more aggressive management for high-grade conditions. While for survival prediction, the decision involved intensified clinical management, such as closer monitoring or consideration of adjuvant therapy, based on the predicted risk.

DCA was performed separately for both classification tasks to assess whether model-guided decisions provide a net clinical advantage over the reference strategies across clinically relevant threshold ranges. All analyses were conducted using predicted class probabilities, without relying on a fixed operating point, ensuring a threshold-agnostic assessment of clinical value.

(1) $$Net\;Benefit\;(p_{t})=\frac{TP}{N}-\frac{FP}{N}\cdot \frac{p_{t}}{1-p_{t}}$$

## Results

### Grade classification

**Figure 1:**
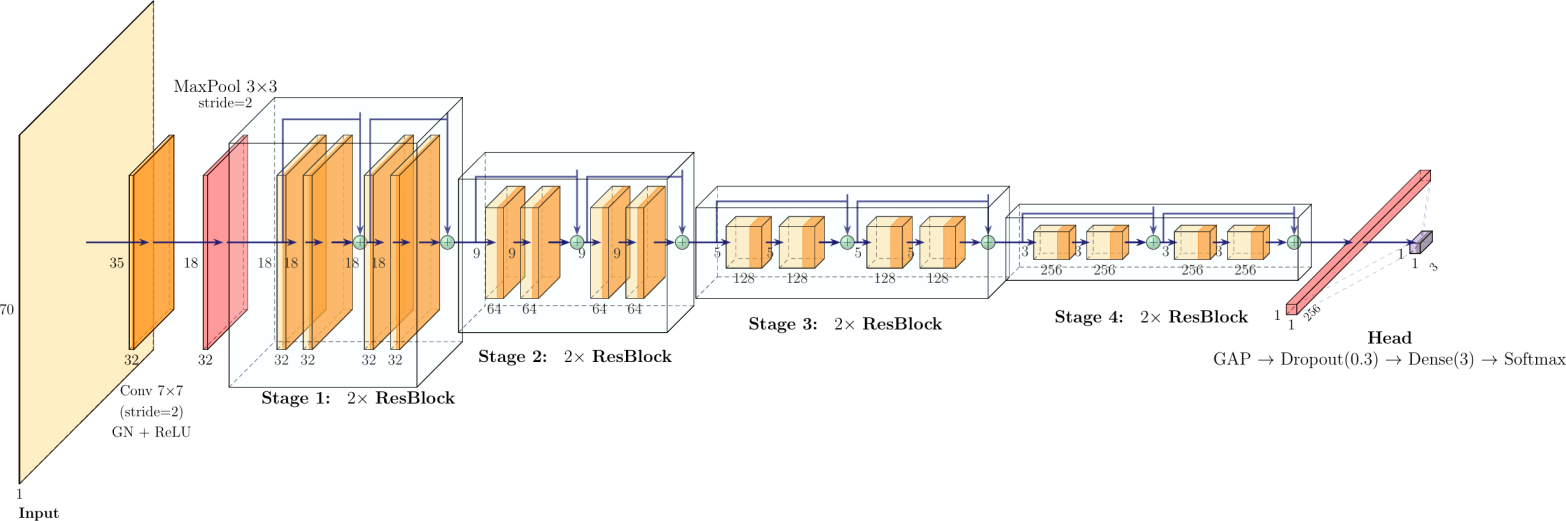
Best-performing model for the grade classification task.

**Figure 2:**
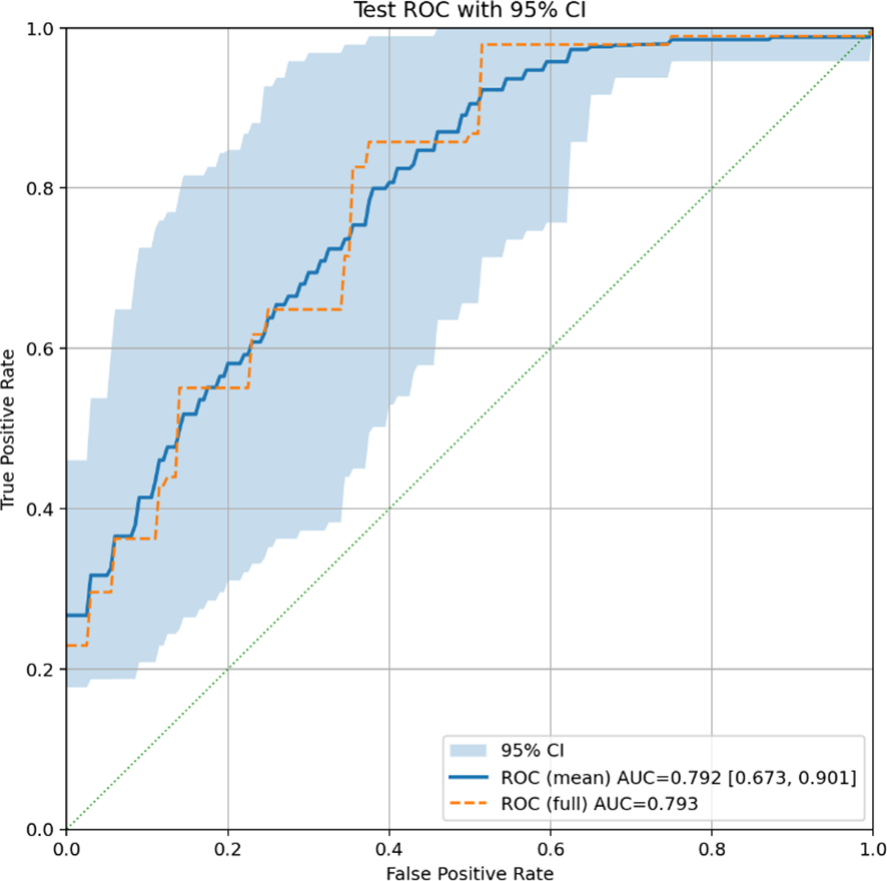
ROC AUC curve for test set, with 95% CI, for the grade classification task.

The best-performing model (**Figure 1**) achieved a Macro-OvR (One vs. Rest) AUC of 0.79 on the held-out test set. Macro OvR AUC is defined in Equation 2 for K classes, and is the standard, most widely used multi-class extension of ROC-AUC. In classification problems with more than two classes, standard metrics are undefined. Instead, weighted metrics are defined and used to evaluate model performance. These are shown in **Table 2**. **Figure 2** shows the Macro OvR ROC-AUC curve for the test set, with a 95% Confidence Interval (CI). For the WHO grade task, the held-out test set consisted of 40 patients, selected with patient-level stratification to preserve grade distribution while preventing an empty grade-3 class.

**Table 2:** Classification metrics for the best-performing model of the grade classification task, evaluated on the held-out test set.

Metric	Value
Macro OvR AUC	0.79
Accuracy	0.60
Weighted Precision	0.76
Weighted Recall (Sensitivity)	0.73
Weighted F1-score	0.74
Support (# test samples)	40

(2) $$Macro\;OvR\;AUC=\frac{1}{K}\sum_{c=1}^{K}AUC(c\;vs\;the\;rest)$$

### OS classification

**Figure 3:**
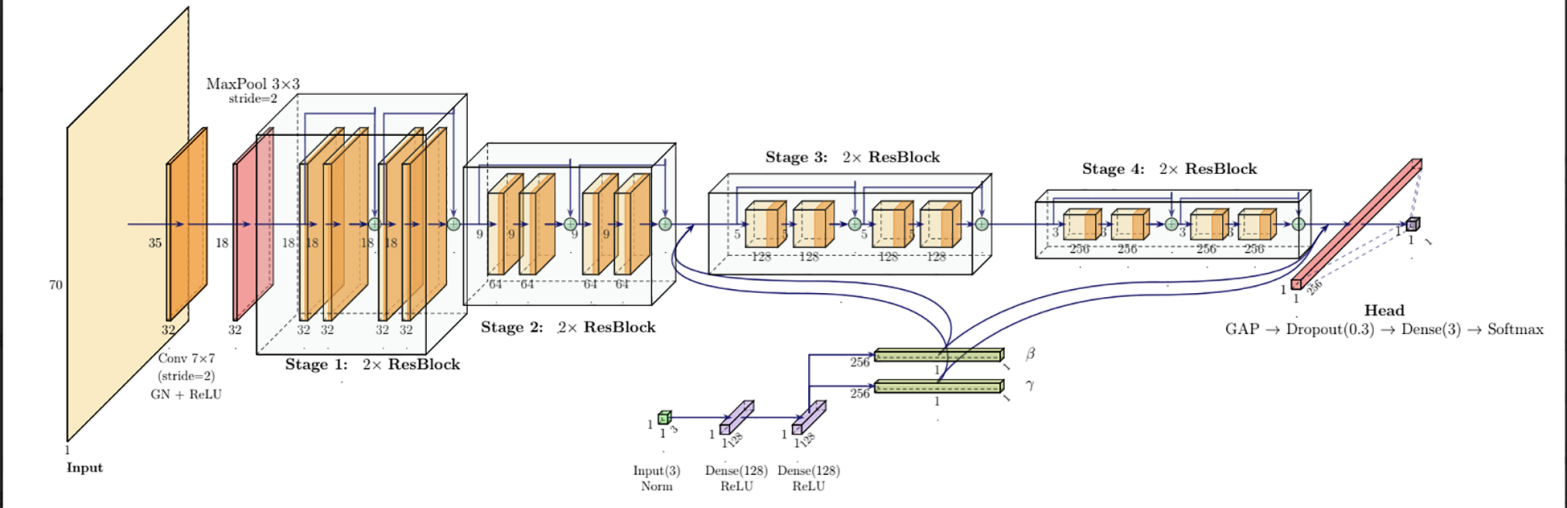
Best-performing model for the OS classification task.

**Figure 4:**
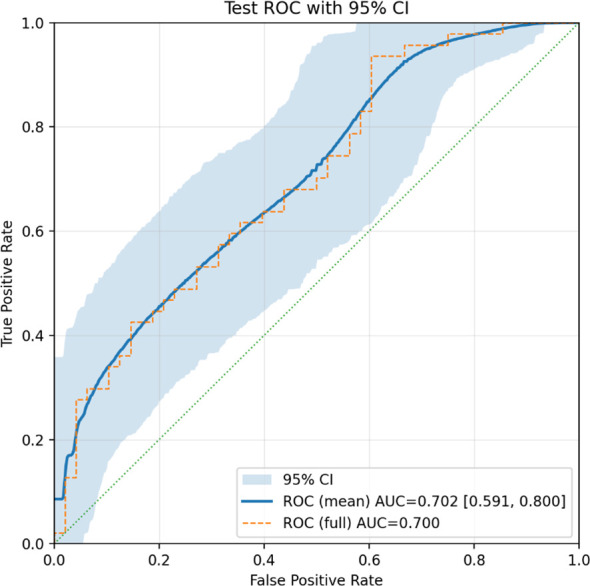
ROC AUC curve for test set, with 95% CI, for the OS classification task.

The best-performing model (**Figure 3**) achieved an AUC of 0.70 on the held-out test set. More binary classification metrics are shown in **Table 3**, for the default probability threshold of 0.5. **Figure 4** shows the ROC-AUC curve for the test set, with a 95% CI.

**Table 3:** Classification metrics for the best-performing model of the OS classification task, evaluated on the held-out test set.

Metric	Value
AUC	0.70
Accuracy	0.66
Precision	0.60
Recall (Sensitivity)	0.94
F1-score	0.73
Support (# test samples)	78

### Explainability

**Figure 5:**
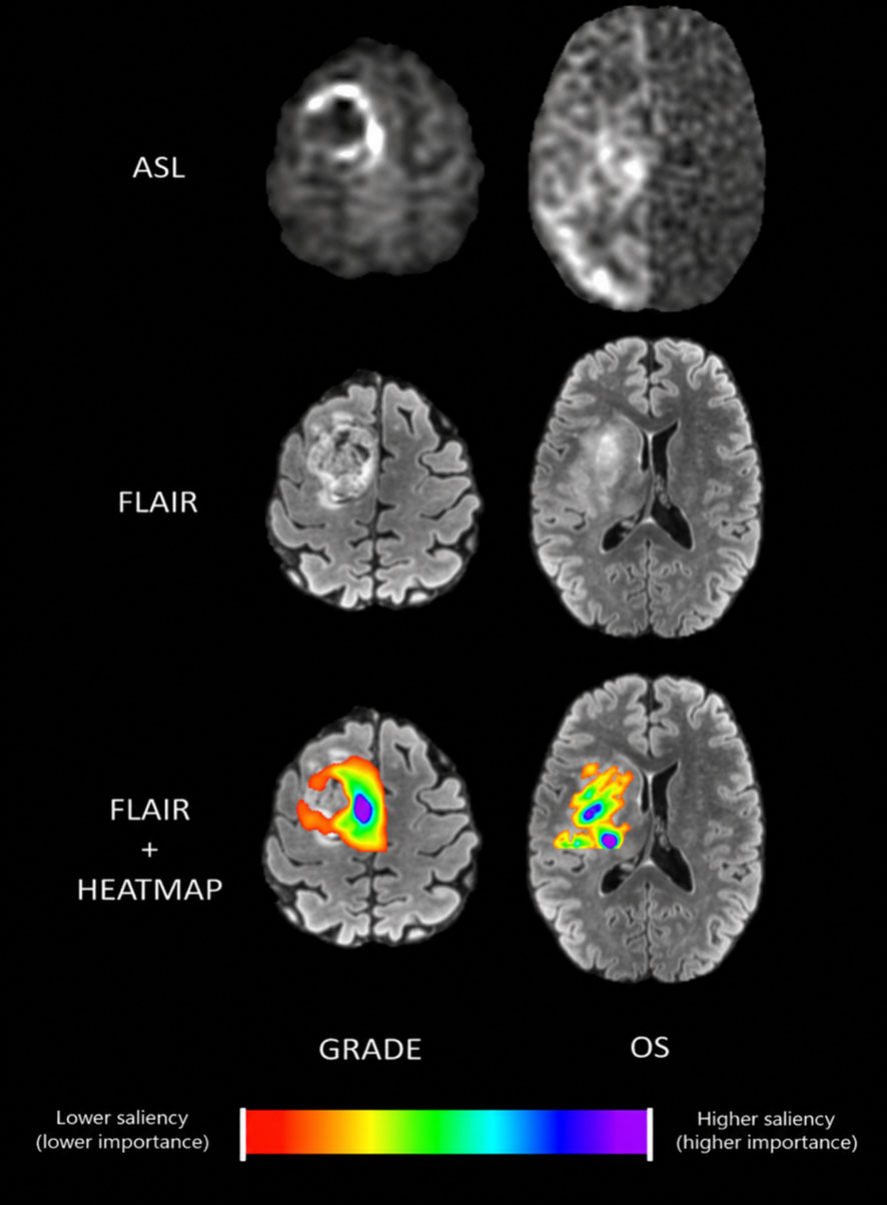
Model attribution maps for glioma grade and overall survival (OS) in two representative patients. For each patient, the same axial slice is shown across modalities. The first column shows ASL images, the second column shows native FLAIR images for anatomical reference, and the third column shows FLAIR images with integrated gradients (IG) saliency overlays. For grade prediction, attribution is predominantly localized within the enhancing tumor core, whereas for OS prediction, attribution extends into the peritumoral region, suggesting the model incorporates microenvironmental features relevant to prognosis. The color scale represents relative attribution intensity, with cooler colors (violet/blue) indicating a higher contribution to the model output.

**Figure 5** presents a slice of the 3D voxel-wise saliency map derived from IG, highlighting the most influential ASL regions for the model output. The map is co-registered and overlaid on the subject’s FLAIR MRI for anatomical reference, as seen in the bottom images, and the corresponding ASL slice is shown at the top. Regions in warmer colors (red/yellow) represent voxels with lower saliency, whereas regions in cooler colors (blue/violet) represent voxels with higher saliency. For the grade classification task, channel contributions for original ASL, global MAD standardization, local standardization, and log-transformed channels are 0.961±0.13, 0.016 ± 0.010, 0.004 ± 0.001 0.019 ± 0.005 respectively. Similarly, for the OS classification task: 0.97±0.01, 0.00±0.00, 0.01±0.00, 0.03±0.01. Overall, the tabular input of the OS model contributed less than 0.1%; among them, age is the dominant tab feature, with sex and EOR contributing roughly equally, but each is less than age. As shown in **Figure 5** (right), the grading model exhibited a compact attribution pattern centered on the tumor core and hyperperfused rim. In contrast, the OS model (**Figure 5**, left) demonstrated a broader attribution distribution extending into surrounding peri-tumoral regions, suggesting that survival prediction may rely on a wider perfusion-defined microenvironmental signature.

### Decision curve analysis

**Figure 6:**
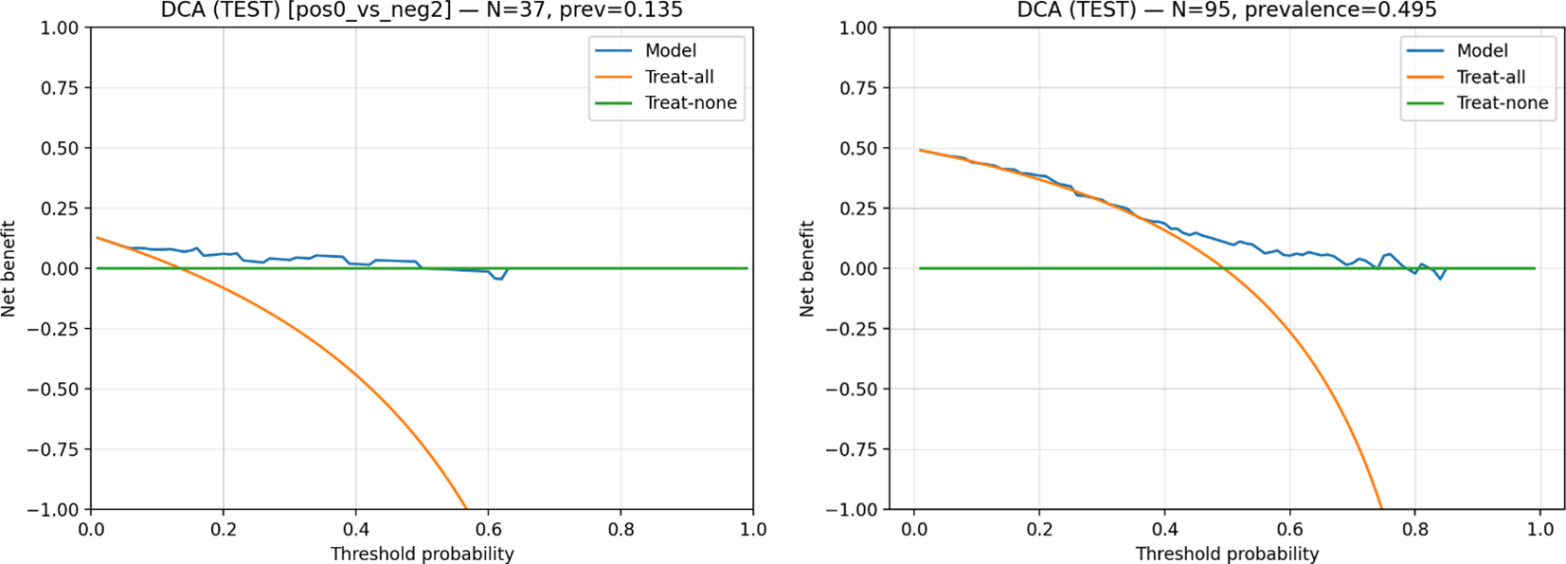
Decision curve analysis for the WHO grade classification task [left] and the OS classification task [right], using test-set probabilities.

Decision curve analysis (**Figure 6**) demonstrated that the proposed models provided a positive net clinical benefit across a range of threshold probabilities for both tasks evaluated.

For the first task (WHO grade II vs IV, N = 37, prevalence = 0.135), the model consistently outperformed both the “treat-all” and “treat-none” strategies across low-to-intermediate threshold probabilities. In this range, the model achieved a positive net benefit, whereas the treat-all strategy rapidly declined and became inferior as the threshold increased. At higher thresholds, the model’s net benefit approached that of the treat-none strategy, indicating diminishing clinical utility when only very high predicted risks are acted on.

For the second task (OS < 12 months Vs OS > 12 months) (N = 78, prevalence = 0.551), the model showed a clear advantage over the treat-none strategy across a broad range of clinically relevant thresholds. Compared with the treat-all strategy, the model yielded comparable net benefit at low thresholds and superior net benefit at intermediate thresholds, where the treat-all approach incurred a substantial penalty due to false-positive decisions. At higher thresholds, net benefit gradually decreased and converged toward zero, reflecting reduced benefit when restricting decisions to only the highest predicted risks.

Overall, decision curve analysis indicates that, for both tasks, model-guided decision making offers a greater net benefit than default strategies across meaningful probability thresholds, supporting the potential clinical usefulness of the proposed approach.

## Discussion

In this study, we developed and validated ASLNet, an explainable DL framework using ASL perfusion for non-invasive glioma grading and survival prediction. Our model shows that perfusion data alone effectively characterizes tumor aggressiveness, supporting the idea that vascular physiology encodes markers of glioma grade and patient outcome. Explainability analysis confirms that ASLNet bases its predictions on meaningful perfusion patterns, overcoming a standard limitation that can undermine clinicians’ trust and enabling visualization of the regions that influenced the decision.

Our model’s behavior closely aligns with known glioma biology. HGG typically exhibit increased neoangiogenesis, higher microvascular density, and heterogeneous cerebral blood flow, whereas LGG often have lower, more homogeneous perfusion (20, 21). The model consistently emphasizes hyperperfused rims and infiltrative peritumoral areas associated with proliferative and angiogenic activity, while assigning negligible importance to necrotic cores and low-signal regions, suggesting that perfusion heterogeneity, rather than structural features, is a key indicator of tumor grade and survival. The contribution analysis indicated that ASLNet primarily depends on the raw ASL perfusion channel, with little impact from standardized and log-transformed data channels. Thus, the model’s predictions are based on the tumor’s intrinsic perfusion features rather than auxiliary channel features, supporting the biological relevance and interpretability of the learned pattern features. Although clinical variables such as age, sex, and EOR are included to improve prediction, they have minimal impact on survival outcomes, reinforcing the link between ASL, grade, and overall survival.

Previous studies using perfusion-based machine learning have shown that advanced hemodynamic imaging can aid in glioma characterization and prognosis, but the research methods vary widely. Earlier work focused on DSC-based radiomics, often combined with structural, diffusion, or contrast-enhanced MRI, and typically targeted a single clinical outcome. For instance, Sudre et al. (2020) applied DSC-MRI radiomics with supervised machine learning to classify gliomas by grade and mutation in a multicenter cohort, reporting a grading accuracy of 53% (22). Additionally, habitat-based methods using DSC perfusion, such as the hemodynamic tissue signature (HTS) introduced by Garcia et al. (2018), have shown that vascular tumor subcompartments identified through perfusion imaging correlate with tumor heterogeneity. These perfusion-defined vascular habitats significantly enhance prognostic models—reducing RMSE from 219 to 184 days and increasing survival stratification accuracy to 78%. This approach also correlates with overall survival and has been incorporated into a publicly available clinical platform, ONCOhabitats, for non-invasive glioblastoma assessment (23, 24). Meanwhile, Park et al. (2024) used diffusion- and perfusion-weighted MRI radiomics, along with clinical data, to improve the prediction of OS in lower-grade gliomas, achieving an AUC of 0.88 (25). Other studies have incorporated multimodal radiomics and neural networks to forecast glioblastoma progression, or have used longitudinal structural-plus-perfusion MRI with machine learning to monitor post-treatment progression (26, 27). Our study differs in that it focuses on an ASL-only, non-contrast, explainable DL framework that assesses both preoperative grading and OS using a single perfusion sequence. The novelty of ASLNet lies not only in introducing perfusion-based DL to glioma imaging, but also in demonstrating the feasibility of an interpretable, perfusion-grounded, ASL-based DL method for assessing two important clinical tasks.

Most previous DL studies for glioma assessment used multiparametric structural MRI, achieving strong results but requiring additional data acquisition, segmentation, and contrast-enhanced imaging. Gutta et al. (2021) applied four MRI modalities (T1, T1CE, T2, FLAIR) using a CNN, achieving an accuracy of approximately 0.87 (28). Yang et al. (2018) developed a transfer-learning CNN on T1 post-contrast images, achieving AUCs of 0.87-0.94. However, due to a smaller sample size, the model was limited in the features it could learn (29). For survival prediction, Jin et al. (2025) combined T1CE radiomics, clinical data, and CNNs, achieving an AUC of around 0.80; their T1CE-only 2D and 2.5D CNN models had AUCs of 0.71 and 0.74, respectively (30).

In contrast, ASLNet uses only ASL perfusion without segmentation and yet achieves encouraging performance for survival and grade prediction, suggesting that a single non-invasive sequence may capture clinically relevant information. Even though the raw metrics are indeed modest, they are acceptable for clinical performance, as the framework’s accuracy relies on a single-modality, perfusion-based model with supporting clinical potential as indicated by DCA. Also, this simplified design reduces preprocessing dependencies and may facilitate future evaluation in broader clinical settings. However, because the present study lacks external validation, the portability and scalability of ASLNet across institutions, scanners, and acquisition protocols remain unproven and should be considered preliminary until confirmed in multi-center studies. Additionally, the 3D CNN architecture is advantageous in that it reduces overfitting and ensures it captures all relevant information, especially in heterogeneous tumors such as glioblastoma, which benefit from 3D modeling (31).

A direct comparison of the two tasks suggests that the grading model currently offers greater potential clinical utility in terms of discrimination, with a Macro OvR AUC of 0.79 despite the relatively small held-out test cohort (n = 40). While this smaller sample size limits robustness and widens uncertainty around the performance estimate, the achieved discrimination remains encouraging for a single-sequence ASL-based framework. In contrast, the OS model achieved a more modest AUC of 0.70 and accuracy of 0.66, indicating only moderate discriminative ability and more limited predictive strength. Accordingly, the survival results should be interpreted primarily as a prognostic proof of concept that supports the biological relevance of ASL-derived perfusion heterogeneity, rather than as a clinically mature predictive tool.

A key highlight of the study was the model’s ability to predict survival groups based solely on ASL imaging. Unlike typical methods that rely on multimodal imaging or genomics, ASLNet detected perfusion patterns beyond the tumor core, including peri-tumoral vasculature, which were associated with survival outcomes, supporting the idea that the tumor microenvironment influences prognosis rather than just tumor size (32, 33). Demographic and surgical factors had little impact, despite their common use in prognostic models. The importance of perfusion features suggests that ASL captures biological information that may overlap with, or even exceed, that of age, sex, or resection extent (34, 35).

ASLNet offers multiple clinical benefits in neuro-oncology by leveraging ASL’s strengths and incorporating features that aid clinical decision-making. ASLNet employs a single, non-invasive perfusion sequence that does not require gadolinium, making it suitable for patients with contrast allergies or renal conditions. In addition to these advantages of ASL, the model provides valuable insights through its architecture and explainability. It produces voxel-level interpretation maps that highlight biologically plausible regions of hyperperfusion and vascular heterogeneity. This allows radiologists to verify the model’s outputs rather than accept opaque predictions. Its capability to perform glioma grading and survival prediction from a single input demonstrates its efficiency and scalability. Its clinical significance was demonstrated in grading and survival tasks, with DCA showing that ASLNet enables risk-based adjustments in diagnostic confirmation or clinical management, outperforming standard strategies at key clinical threshold probabilities. However, this benefit is threshold-dependent and remains modest. The grading model shows its main incremental value in the intermediate thresholds (approximately 30-75%), whereas the OS model is most useful in a narrower lower-to-intermediate range (approximately 5-50%). Outside these intervals, net benefit approaches default strategies, indicating that clinical usefulness depends on the selected decision threshold. Therefore, ASLNet results may serve as a supplement to institutional tumor boards, offering additional insights for decisions such as treatment escalation, follow-up duration, and surgical management.

Our study has several significant strengths. We present the first explainable 3D ResNet framework that simultaneously performs glioma grading and survival prediction using only a single ASL perfusion sequence. This demonstrates that perfusion-based vascular information alone can support these crucial clinical tasks, which is one of the main advantages of our study. The model addresses ASL’s low SNR and low resolution by leveraging residual learning and a standardized 3D ROI, eliminating the need for tumor segmentation. ASLNet also incorporates clinical variables and provides voxel-level interpretability that aligns with known tumor physiology, thereby enhancing clinical trust. At the same time, DCA further supports the clinical benefit of our framework.

However, the study has some important limitations. First, it lacks external multicenter validation, which may impact the models’ generalizability. Second, missing post-treatment data on chemotherapy, radiotherapy, and Karnofsky Performance Status could improve survival predictions. Third, a direct comparison of neuroradiologists’ and neuropathologists’ performance, or of established clinical nomograms, was not performed to assess clinical utility, which remains essential for evaluating its value. While the DCA showed clear clinical benefit, it should be considered preliminary pending further validation. Future research should validate ASLNet on different scanners and clinical settings, including longitudinal and treatment data, to ensure its reliability. Lastly, assessing whether pre-treatment ASL can predict responses to anti-angiogenic therapies such as bevacizumab is a valuable direction for enhancing the clinical applications of ASLNet.

In summary, this study presents ASLNet, an explainable DL framework trained solely on ASL perfusion MRI to predict glioma grade and survival. The findings support the feasibility of using perfusion-based imaging to capture biologically relevant vascular and microenvironmental patterns associated with tumor behavior. Its voxel-level interpretability may enhance clinical transparency and confidence in model outputs. However, ASLNet should currently be regarded as a promising proof of concept, as the study lacks external validation and longitudinal, multi-center testing. Accordingly, future work should focus on rigorous validation across institutions, scanners, acquisition protocols, and patient populations before clinical translation can be considered.

## Data Availability

Publicly available datasets were analyzed in this study. This data can be found here: The dataset analyzed in this study are publicly available. Imaging data and associated clinical annotations were obtained from the University of California San Francisco Preoperative Diffuse Glioma MRI (UCSF-PDGM) dataset, available through The Cancer Imaging Archive (TCIA). Repository: The Cancer Imaging Archive (TCIA) Dataset Name: UCSF-PDGM (Version 5) DOI/Accession: https://doi.org/10.7937/tcia.bdgf-8v37 Direct dataset link: https://www.cancerimagingarchive.net/collection/ucsf-pdgm/.
