# Supplementary material for: ASLNet: an explainable deep learning framework for glioma grading and survival prediction
Source: Front Oncol. 2026 May 18;16:1818663. doi: 10.3389/fonc.2026.1818663 (PMC13223080; doi:10.3389/fonc.2026.1818663)
Supplement: Supplementary file 1 [file Table1.docx]

## Scanners and Acquisition Parameters

## Preoperative MRI was obtained from the UCSF-PDGM dataset using a standardized 3T GE Discovery MR750 protocol that included 3D arterial spin labeling (ASL) among the preoperative sequences. The public TCIA dataset documentation provides scanner-level protocol information but does not report the complete vendor-specific ASL pulse sequence parameterization (e.g., CASL/pCASL implementation, TR, TE, post-labeling delay, or labeling duration), which is an inherent limitation of this retrospective public dataset.

## Training and Testing Split Statistics

| **Metric** | **Train-CV pool** | **Test set** | **p-value** | **Test performed** |
| --- | --- | --- | --- | --- |
| Patients (n) | 312 | 78 | - | - |
| Death | 174 | 43 | - | - |
| Survival | 138 | 35 | - | - |
| Prevalence (death) | 0.558 | 0.551 | 1.0000 | Fisher’s exact |
| OS mean ± SD (months) | 16.77 ± 15.21 | 16.92 ± 15.24 | 0.6606 | Mann-Whitney U |
| Age | - | - | 0.7180 | Mann-Whitney U |
| Sex | - | - | 0.6074 | Fisher’s exact |
| EOR | - | - | 0.9795 | $\chi^{2}$ |

Table 1: Detailed class distributions within the TRAIN–CV pool and the held-out TEST sets for the OS and WHO grade prediction tasks.

## Detailed Preprocessing Steps

Preprocessing comprises four distinct steps: ROI definition, feature channel creation, data augmentation, and splitting to train and test sets. The first three steps are identical for both the grade and OS classification tasks, and only ASL volumes were used, with no additional modalities or segmentation files. Also, all volumes were initially of size 240 × 240 × 155, and all voxels were isotropic, of size 1 × 1 × 1 $=1 mm^{3}$. First, for each ASL volume, the central tumor region is approximated by selecting the top 0.01% brightest voxels. Then, their centroid is computed, and a 70 × 70 × 70 $mm^{3}$ cubic ROI is formed around it. After visually inspecting the regions, it was confirmed that the tumor was mostly contained within them, along with surrounding tissue and, in some cases, a few background voxels. To deal with the latter, an approximate brain mask was obtained by identifying a per-scan background volume threshold ($v_{f}$), isolating subregions within the cube that, if present, constituted a percentile (around 0.5%) of uniform intensity near zero. These subregions were clearly part of the background and were excluded from subsequent statistical computations in the preprocessing pipeline. Secondly, three additional ASL-derivative feature channels were added to accompany the original ASL volumes in the input vector. The first-derivative channel is produced by robustly standardizing the ASL volume, excluding the background region, using Eq. 1, where 1.4826 is the Gaussian consistency factor [1]. MAD stands for Medial Absolute Deviation and is defined in[2]. The second-derivative channel is produced by applying local standardization to 15 × 15 × 15 subregions of the original ASL volume.

In contrast, the third one is derived from applying the transformation in Eq. 2 to the original ASL volume, $v_{f}$ with the intensity threshold below which it was previously determined that voxels belong to the background. The following preprocessing step comprised 3D data augmentation, using random flips along any subset of axes, small rotations $\pm10^{\circ}$ around random planes, elastic deformation (smooth random displacement fields), and Rician noise. The latter is chosen instead of Gaussian noise, as MRI images do not contain Gaussian noise. Instead, the scanner reconstructs data by taking the magnitude of complex-valued measurements, and the originally Gaussian noise components combine into a Rician distribution, which is positively biased at low signal levels. This specific type of bias artificially elevates low-intensity voxels, especially in low-SNR modalities like ASL, so robust preprocessing methods like MAD-based normalization, masking, and log transforms are required[3]. The fourth and final preprocessing step differs between the grade and OS tasks. For grade preprocessing, WHO grades II, III, and IV are treated as separate classes; thus, the problem is formulated as a ternary classification task. The test set consists of all samples from 40 distinct and randomly selected patients, whereas the remaining 431 patients (455 scans) were assigned to the train-CV pool are assigned to the train set. For OS preprocessing, the task is formulated as binary classification, with class 1 indicating mortality within 12 months and class 0 indicating no mortality within 12 months.

| $v_{i}^{'}=\frac{v_{i}-median}{1.4826\cdot MAD}$ | Eq. 1 |
| --- | --- |
| $v_{i}^{'}=\log(1+max(v_{i}, v_{f}))$ | Eq. 2 |

## Explainability

Integrated Gradients (IG) is used to produce a 3D saliency map of each voxel’s importance to the final classification outcome, as well as a contribution percentage for each of the four input channels.

The saliency map employed a custom version of IG with smoothing, denoising, robust normalization, and affine reconstruction. For each sample, the target class is selected as the most probable one, and a baseline volume of all-zero intensities is created, shaped like the input volumes, representing the absence of signal. Then, a composite volume is designed as a linear combination of the original and the baseline volume’s voxel intensities, according to Eq. 3. For 128 different values of the parameter $a$, the gradient of class scores with respect to the composite volume is computed, using TensorFlow’s GradientTape. Gradients are then averaged $\left( \bar{g} \right)$ for each value of $a$, and the final attribution score for each voxel is calculated using Eq. 4. Smoothing is performed by perturbing $x_{original}$ with low-variance Gaussian noise for $N_{smooth}$ times and averaging the resulting gradients, effectively mitigating the outlier effect. Spatial denoising is performed by summing the absolute gradient values per channel, to produce a single-channel 3D saliency map. Lastly, gradient values are normalized using the 1^st^ and 99.5^th^ percentiles, as in Eq. 5, aiming to suppress irrelevant and weak attributions before final presentation to the viewer.

Channel contributions are also based on IG, and are produced according to Eq. 6, by averaging the IG magnitude over all three spatial dimensions for a channel and normalizing this value by dividing by the spatial and channel-wise average.

| $x\left( a \right)=x_{baseline}+a(x_{original}-x_{baseline})$ | Eq. 3 |
| --- | --- |
| $IG\left( x \right)=\left( x_{original}-x_{baseline} \right)\odot\bar{g}, \bar{g}=\frac{1}{N_{a}}\sum_{k=1}^{N_{a}} \frac{\partial Model\left( x\left( a_{k} \right) \right)}{\partial x_{original}}$ | Eq. 4 |
| $IG_{norm}=\frac{clip(IG-p_{1})}{p_{99.5}-p_{1}}$ | Eq. 5 |
| $w_{c_{i}}=\frac{\sum_{x,y,z} \left\vert IG(x, y, z,c_{i}) \right\vert}{\sum_{x,y,z,c} \left\vert IG(x, y, z,c) \right\vert}$ | Eq. 6 |

## Detailed Grade Classification Architecture

This section presents the general architecture developed for the grade classification task, while the specific hyperparameter values (e.g. number of layers and neurons per layer) are selected in the hyperparameter optimization process by Optuna, described in a separate section. A general diagram is shown in Figure 1 of the main manuscript.

Input size is $(B, D, H, W, C)$ where $B$ is the batch size, $D=H=W=70$ is depth, height and width respectively, whereas $C=4$ the number of channels per sample.

Input first passes through the step section of the model, which consists of a 3D convolutional layer with a kernel size of 7 and a stride of 2, roughly halving the spatial resolution. Then comes a customized group normalization layer, which splits channels in groups and performs normalization according to Eq. 7, using group mean and standard deviation, with β and γ being trainable parameters that allow the network to avoid the mean strictly equaling 0 and the standard deviation strictly equaling 1, which would limit representational capacity. Afterwards, the output is passed through a ReLU activation and a 3D max pooling layer with a pool size of 3 and a stride of 2.

| $y=\gamma\hat{x}+\beta, \hat{x}=\frac{x-\mu}{\sqrt{\sigma^{2}+\varepsilon}}, \varepsilon={10}^{-5}$ | Eq. 7 |
| --- | --- |

. After the stem follow four residual stages. The number of blocks per stage, stage type (bottleneck or standard for stages 2 to 4), dropout rate, and initial filters are configurable and determined with hyperparameter optimization. Each block consists of a convolutional layer with a kernel size of three and a configurable stride, followed by a group normalization layer, ReLU activation, a convolutional layer with a kernel size of 3 and a stride of 1, and finally another group normalization layer. The shortcut path is optional and, when present, consists of a convolutional layer with kernel size 1 and a stride that matches the dimensions of the added representations, followed by a group normalization layer. In shortcut blocks, the merging part consists of an addition layer, ReLU activation and 3D spatial dropout.

The first residual stage is unique in three ways. Firstly, the first block of the first stage has spatial stride of 1 and follows standard logic, devoid of a bottleneck, whereas first blocks of later stages employ spatial downsampling with stride 2. Secondly, stage 1 does not double the number of filters before processing, operating as a shallow feature extractor on filters determined by the stem, and, as a consequence, it does not need a projection in its first block for compatibility with the shortcut. Stages 2 to 4 also employ a configurable number of blocks, and at the transition between stages, the number of filters is doubled. Overall, the residual stage progressively spatially downsamples the volume and simultaneously increases channel depth to learn more complex patterns while maintaining gradient flow stability through skip connections.

The residual stages are followed by a classification head consisting of a global average pooling layer, which averages over all spatial dimensions independently for each channel, resulting in an output of shape $(B, C_{out})$. This output is then passed to a dropout layer and, finally, to a Softmax-activated fully connected layer, which returns class-wise probabilities.

## Detailed OS Classification Architecture

## The OS binary classification task utilizes a similar architecture, shown in Figure 3 of the main manuscript, to the grade classification task, with the major difference being the integration of tabular data to the ResNet base. This is accomplished using a small Multi-Linear Perceptron (MLP) which receives a vector containing age, sex and EOR, normalizes it and passes is through a fully connected network with dropout. Then, the resulting representation is passed through two different, customizable fully connected, tanh-activated layers, which output conditioning vectors $\boldsymbol{\gamma}_{\boldsymbol{c}}$ and $\boldsymbol{\beta}_{\boldsymbol{c}}$, respectively. Integration is achieved using Feature-wise Linear Modulation (FiLM)[4], on the output of each block of the last three residual stages, namely applying the linear transformation of Eq. 8 voxel-wise to each channel of a block’s output by matching conditioning vector elements to their corresponding channels. This enables parameter-efficient, stable, and learnable modulation of tabular data within the ResNet architecture, allowing clinical covariates to influence image representations at deeper network stages dynamically. In this work, FiLM is applied only to the last three stages because early convolutional layers tend to learn low-level anatomical features that should remain undistorted and image-driven. At the same time, higher-level semantic representations are meaningfully influenced by tabular variables, which are typically found in later stages.

| $FiLM\left( x \right)=\gamma_{c}x+\beta_{c}$ | Eq. 8 |
| --- | --- |

## Detailed Grade Classification Hyperparameter Optimization

Hyperparameter optimization is performed using Optuna’s TPE sampler[5]. The types and ranges of the optimized hyperparameters are shown in Table 2. Specifically, they comprise the learning rate for the Adam optimizer, its weight decay, the number of groups for group normalization, the presence or absence of a bottleneck in the residual blocks, of the stem output filters, spatial and regular dropout rate, early stopping patience, learning rate plateauing patience, and label smoothing factor for the categorical cross-entropy loss.

| **Hyperparameter** | **Type and range** | **Optimized Value** |
| --- | --- | --- |
| Optimizer learning rate | $\left[ {10}^{-4},{10}^{-3} \right] (log-scale)$ | $1.4\cdot{10}^{-4}$ |
| Optimizer weight decay | $\{0, {10}^{-7},{3\cdot10}^{-7},{10}^{-6},{3\cdot10}^{-6},{10}^{-7},{3\cdot10}^{-7},{10}^{-5},{3\cdot10}^{-5},{10}^{-4},{3\cdot10}^{-4}\}$ | ${10}^{-7}$ |
| Group normalization groups | $\left\{ 4, 8, 16, 32 \right\}$ | $32$ |
| Bottleneck | $\{True, False\}$ | $False$ |
| Initial filters | $\left\{ 16, 24, 32, 48 \right\}$ | $32$ |
| Spatial and head dropout rates | $\left\{ 0.0, 0.1, 0.2, 0.3, 0.4 \right\}$ | $0.3$ |
| Early stopping patience | $\left[ 10, 22 \right] (integers)$ | $15$ |
| Plateauing patience | $\left[ 2, 5 \right] (integers)$ | $3$ |
| Label smoothing factor | $\left[ 0.0, 0.12 \right]$ | $0.0031$ |

Table 2: Hyperparameter search space for the grade classification task.

## Detailed OS Classification Hyperparameter Optimization

Hyperparameter optimization is also performed using Optuna’s TPE sampler. The types and ranges of the optimized hyperparameters are shown in Table 3. Specifically, they comprise the learning rate for the Adam optimizer, its weight decay, the number of groups for group normalization, the presence or absence of bottleneck in the residual blocks, of the stem output filters, spatial and regular dropout rate, early stopping patience, learning rate plateauing patience, hidden width of the FiLM MLP and FiLM dropout rate.

| **Hyperparameter** | **Type and range** | **Optimized Value** |
| --- | --- | --- |
| Optimizer learning rate | $\left[ {10}^{-4},{10}^{-3} \right] (log-scale)$ | $4.4\cdot{10}^{-4}$ |
| Optimizer weight decay | $\{0, {10}^{-7},{3\cdot10}^{-7},{10}^{-6},{3\cdot10}^{-6},{10}^{-7},{3\cdot10}^{-7},{10}^{-5},{3\cdot10}^{-5},{10}^{-4},{3\cdot10}^{-4}\}$ | ${10}^{-7}$ |
| Group normalization groups | $\left\{ 4, 8, 16, 32 \right\}$ | $32$ |
| Bottleneck | $\{True, False\}$ | $False$ |
| Initial filters | $\left\{ 16, 24, 32, 48 \right\}$ | $32$ |
| Spatial and head dropout rates | $\left\{ 0.0, 0.1, 0.2, 0.3, 0.4 \right\}$ | $0.1$ |
| Early stopping patience | $\left[ 10, 22 \right] (integers)$ | $16$ |
| Plateauing patience | $\left[ 2, 5 \right] (integers)$ | $4$ |
| Hidden FiLM size | $\left\{ 64, 128 \right\}$ | $128$ |
| FiLM dropout rate | $\{0.0, 0.1, 0.2\}$ | $0.0$ |

Table 3: Hyperparameter search space for the OS classification task.

## Checklist for Artificial Intelligence in Medical Imaging (CLAIM)

| Section / Topic | No. | Item | Page / Line | No | NA |
| --- | --- | --- | --- | --- | --- |
| TITLE / ABSTRACT |  |  |  |  |  |
|  | **1** | Identification as a study of AI methodology, specifying the category of technology used (e.g., deep learning) | **1/25** |  |  |
| ABSTRACT |  |  |  |  |  |
|  | **2** | Summary of study design, methods, results, and conclusions | **1/22** |  |  |
| INTRODUCTION |  |  |  |  |  |
|  | **3** | Scientific and/or clinical background, including the intended use and role of the AI approach | **2/66** |  |  |
|  | **4** | Study aims, objectives, and hypotheses | **2/81** |  |  |
| METHODS |  |  |  |  |  |
| *Study Design* | **5** | Prospective or retrospective study | **3/94** |  |  |
|  | **6** | Study goal | **2/86** |  |  |
| *Data* | **7** | Data sources | **3/94** |  |  |
|  | **8** | Inclusion and exclusion criteria | **3/98** |  |  |
|  | **9** | Data pre-processing | **3/106** |  |  |
|  | **10** | Selection of data subsets | **3/122** |  |  |
|  | **11** | De-identification methods |  |  | **X** |
|  | **12** | How missing data were handled |  |  | **X** |
|  | **13** | Image acquisition protocol | **3/100** |  |  |
| *Reference Standard* | **14** | Definition of method(s) used to obtain reference standard |  |  | **X** |
|  | **15** | Rationale for choosing the reference standard |  |  | **X** |
|  | **16** | Source of reference standard annotations | **2/93** |  |  |
|  | **17** | Annotation of test set | **2/93** |  |  |
|  | **18** | Measures of inter- and intra-rater variability of features described by the annotators |  |  | **X** |
| *Data Partitions* | **19** | How data were assigned to partitions | **5/172** |  |  |
|  | **20** | Level at which partitions are disjoint | **5/171** |  |  |
| *Testing Data* | **21** | Intended sample size | **5/209** |  |  |
| *Model* | **22** | Detailed description of model | **4/144** |  |  |
|  | **23** | Software libraries, frameworks, and packages | **3/102** |  |  |
|  | **24** | Initialization of model parameters | **5/173** |  |  |
| *Training* | **25** | Details of training approach | **5/168** |  |  |
|  | **26** | Method of selecting the final model | **5/204** |  |  |
|  | **27** | Ensembling techniques |  |  | **X** |
| *Evaluation* | **28** | Metrics of model performance | **5/213** |  |  |
|  | **29** | Statistical measures of significance and uncertainty | **5/208** |  |  |
|  | **30** | Robustness or sensitivity analysis |  |  | **X** |
|  | **31** | Methods for explainability or interpretability | **4/128** |  |  |
|  | **32** | Evaluation on internal data | **5/213** |  |  |
|  | **33** | Testing on external data |  |  | **X** |
|  | **34** | Clinical trial registration |  |  | **X** |
| *RESULTS* |  |  |  |  |  |
| *Data* | **35** | Numbers of patients or examinations included and excluded | **3/93** |  |  |
|  | **36** | Demographic and clinical characteristics of cases in each partition | **4/127** |  |  |
| *Model performance* | **37** | Performance metrics and measures of statistical uncertainty | **5/208** |  |  |
|  | **38** | Estimates of diagnostic performance and their precision | **5/228** |  |  |
|  | **39** | Failure analysis of incorrect results |  |  | **X** |
| *DISCUSSION* |  |  |  |  |  |
|  | **40** | Study limitations | **7/327** |  |  |
|  | **41** | Implications for practice, including intended use and/or clinical role | **7/309** |  |  |
| *OTHER INFORMATION* |  |  |  |  |  |
|  | **42** | Provide a reference to the full study protocol or to additional technical details |  |  | **X** |
|  | **43** | Statement about the availability of software, trained model, and/or data | **2/88** |  |  |
|  | **44** | Sources of funding and other support; role of funders |  |  | **X** |

Table 4: Compliance with CLAIM Checklist

References
1. Hampel FR. The Influence Curve and its Role in Robust Estimation. Journal of the American Statistical Association. 1974;69:383–93. https://doi.org/10.1080/01621459.1974.10482962

2. Rousseeuw PJ, Croux C. Alternatives to the Median Absolute Deviation. Journal of the American Statistical Association. 1993;88:1273–83. https://doi.org/10.1080/01621459.1993.10476408

3. Sijbers J, Den Dekker AJ, Van Audekerke J, Verhoye M, Van Dyck D. Estimation of the Noise in Magnitude MR Images. Magnetic Resonance Imaging. 1998;16:87–90. https://doi.org/10.1016/S0730-725X(97)00199-9

4. Perez E, Strub F, Vries H de, Dumoulin V, Courville A. FiLM: Visual Reasoning with a General Conditioning Layer [Internet]. arXiv; 2017 [cited 2025 Dec 8]. https://doi.org/10.48550/arXiv.1709.07871

5. Akiba T, Sano S, Yanase T, Ohta T, Koyama M. Optuna: A Next-generation Hyperparameter Optimization Framework [Internet]. arXiv; 2019 [cited 2025 Sept 14]. https://doi.org/10.48550/arXiv.1907.10902
